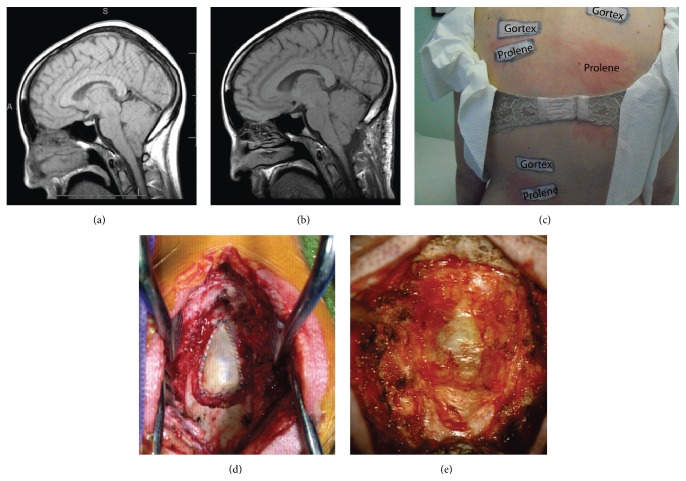# Allergy to Prolene Sutures in a Dural Graft for Chiari Decompression

**DOI:** 10.1155/2015/583570

**Published:** 2015-12-21

**Authors:** Iahn Cajigas, S. Shelby Burks, Joanna Gernsback, Lauren Fine, Baharak Moshiree, Allan D. Levi

**Affiliations:** ^1^Department of Neurological Surgery and the Miami Project to Cure Paralysis, University of Miami Miller School of Medicine, Miami, FL 33136, USA; ^2^Division of Pulmonary, Allergy, Critical Care, and Sleep Medicine, University of Miami Miller School of Medicine, Miami, FL 33136, USA; ^3^Division of Gastroenterology, University of Miami Miller School of Medicine, Miami, FL 33136, USA

## Abstract

Allergy to Prolene suture is exceedingly rare with only 5 cases reported in the literature. There have been no such cases associated with neurosurgical procedures. Diagnosis is nearly always delayed in spite of persistent symptomatology. A 27-year-old girl with suspected Ehlers-Danlos, connective tissue disorder, underwent posterior fossa decompression for Chiari Type 1 malformation. One year later, the patient presented with urticarial rash from the neck to chest. Cerebrospinal fluid and blood testing, magnetic resonance imaging, and intraoperative exploration did not suggest allergic reaction. Eventually skin testing proved specific Prolene allergy. After suture material was removed, the patient no longer complained of pruritus or rash. This single case highlights the important entity of allergic reaction to suture material, namely, Prolene, which can present in a delayed basis. Symptomatology can be vague but has typical allergic characteristics. Multidisciplinary approach is helpful with confirmatory skin testing as a vital part of the workup.

## 1. Introduction

Allergic reaction to polypropylene, Prolene (Ethicon, Somerville, New Jersey, USA), suture material is a rare but reported entity. Here we present a case of such a reaction to Prolene sutures on a dural allograft used during posterior fossa decompression for Chiari Type 1 malformation. The diagnosis was made with confirmatory skin testing. The patient's associated symptoms resolved after removal of suture material.

## 2. Case Presentation

A 27-year-old woman with a history of Ehlers-Danlos, connective tissue disorder, presented with a several-year history of headaches, bilateral upper and lower extremity pain, and neck pain. She complained of weakness to her upper extremities, left greater than right. She also reported numbness of her left arm with paresthesias to her feet bilaterally. She had balance difficulty with periodic moments of dizziness.

On physical exam, she had full strength on the right but had mild left-sided weakness (4/5) in the deltoid, finger flexors, hand intrinsic, quadriceps, and tibialis anterior. She had decreased sensation to light touch and pinprick in the left upper and lower extremities. Coordination and cerebellar testing revealed suggested mild dysfunction, and a subtle left pronator drift was elicited. Magnetic resonance imaging (MRI) done at that time demonstrated a significant Chiari Type 1 malformation with tonsillar descent of approximately 13 to 14 mm and significant obstruction of the foramen magnum (Figure 1(a)). The fourth ventricle appeared normal and there was no evidence of a syrinx. Due to the constellation of neurological symptoms and imaging evidence of a Chiari Type 1 malformation, the patient was taken for posterior fossa craniectomy with C1 laminectomy, an intradural exploration, bipolar of the cerebellar tonsils, and duraplasty using a cadaveric dural graft sutured with 5-0 running Prolene stitches.

In the ensuing two months, the patient required two separate wound revisions. In each case, there was no evidence of infection or cerebrospinal fluid (CSF) leak. Cultures were negative and underlying tissue appeared healthy (Figure 1(d)). The problems with wound healing were attributed to the patient's connective tissue disorder. Of note there was investigation into the possibility of allergic reaction to foreign material placed at the time of initial surgery. Multiple lumbar punctures demonstrated no abnormal elevation in eosinophils with normal opening pressure. Similarly, the peripheral eosinophil count was not elevated. Over the ensuing months, the patient experienced debilitating allergic symptoms including significant pruritic, urticarial rash centered on the nape of the neck, at the site of her cranial surgery. She concurrently developed severe gastroparesis confirmed with gastric emptying study. She required lengthy hospitalizations with total parental nutrition and eventual placement of jejunostomy tube. The impaired gastric motility was, after extensive workup, deemed to be caused by large quantities of antihistamine medications.

Given the lack of inflammatory cells in the CSF and peripheral blood along with the normal appearance of the graft and wound bed on revision surgery, it was difficult to imagine a rejection of the cadaveric dural allograft. The possibility of Prolene suture rejection was then considered and preliminary testing was carried out in hospital with placement of subcutaneous Prolene stitches. Reaction was seen with erythema and pruritus within 24 hours after placement. The patient was referred to an outside allergist and skin testing to suture material was undertaken. In this case, skin testing involved patch testing where suture material was adhered with skin tape and then patient returned to office in 48 hours. This 48-hour patch test was grossly positive and is detailed in Figure 1(c). Further in-office patch testing also demonstrated an immediate allergy to Prolene seen after only 30 minutes of application to skin. With this information, the patient was then taken back to the operating room for removal of Prolene stitches 14 months after the original surgery. Intraoperatively the dural patch was reexposed, as was the Prolene stitch along its border. The Prolene suture material was encased in inflammatory and scar tissue but could be removed completely (Figure 1(e)). No new dural sutures were required, as the patch-graft had incorporated well. The overlying tissues were closed primarily. The patient did require one wound revision due to dehiscence. After the neck wound healed, the patient was seen in follow-up at 4 months postoperatively and no longer complained of neck itching or rash.

## 3. Discussion

Polypropylene, otherwise known as Prolene (trade name from Ethicon), is a nonabsorbable suture. It is a synthetic monofilament with fair tensile strength and minimal tissue reactivity, in general [1]. Allergic reaction to Prolene sutures is very rare, but there have been cases reported.

The first such case, in 1986, describes an allergic reaction in the eye months after cataract surgery. Authors detail conjunctival erythema akin to giant papillary conjunctivitis that abated after removal of a Prolene suture [2]. A second report, many years later in 2003, details a Prolene suture allergy that was confirmed by skin-allergen testing [3]. In this case authors had a suspicion for suture allergy given a history of wound dehiscence with no other clear cause and outpatient allergy testing confirmed a specific Prolene allergy via patch testing confirmed at 2 and 4 days. Another case was presented in 2006 when dermatologists described a 47-year-old woman who developed new-onset eczematous dermatitis characterized by rash and extreme pruritus covering much of her body [4]. This reaction was associated with excision of a benign tumor on the thigh and deep closure using Prolene sutures 6 months before. Symptoms rapidly resolved following removal of the suture material. No confirmatory testing was performed. Again in 2006, Chung et al. presented a case of a retained Prolene suture fragment in the eye causing what was thought to be conjunctival malignancy [5]. In this case, authors elicited a history of blepharoptosis surgery about 3 years before and on careful dissection in the operating room a small Prolene fragment was found and removed. Symptoms rapidly resolved after this. Finally, the most recent case in the literature was described in 2013 where a patient developed a nasal tip abscess 2 years after rhinoplasty [6]. Cultures at the time of revision surgery were negative, and the patient's symptoms resolved with removal of the Prolene stitches. Specific Prolene allergy was then confirmed by skin testing. Interestingly, in this case patch-testing was negative but when the Prolene was sutured into the skin, inflammatory reaction was seen at both 2 and 3 days.

Allergy to polypropylene, that is, Prolene, stitches is obviously rare. Since introduction of this material, there have been a total of five reported cases [2–6]. In two of these, skin-allergen testing confirmed specific Prolene allergy. It is important to keep in mind that the coloring agent used in suture material can also illicit an allergic reaction [6]. It does not appear that this has been teased out in any of the above-mentioned cases. The timeline for treatment, that is, removal of suture, in these cases was often delayed due to either difficulty in diagnosis or insidious presentation. The timing of reaction to the Prolene sutures in our case was rapid and reaction was seen within 30 minutes. In the two cases where skin testing was used, reaction was seen at least within 48 hours. The mechanism believed responsible for our and those cases reported is a delayed-type hypersensitivity reaction, mediated by T-cells responding to antigen on the suture material.

The present case highlights an important consideration when using nonabsorbable, synthetic sutures. This is especially true in neurosurgical procedures where removal of deeply imbedded sutures can be both technically difficult and inherently morbid.

## 4. Conclusion

Allergy to polypropylene, also known as Prolene, sutures is an entity to consider in cases of wound dehiscence without infectious etiology or in delayed allergic-type reaction at the wound site or elsewhere. Skin-allergen testing appears to be a reliable method for diagnosis and confirmation. Inclusion of specialists in allergy medicine and/or dermatology is recommended.

## Figures and Tables

**Figure 1:**